# Endoscopic Treatment of Sphenoid Sinus Mucocele: Case Report and Surgical Considerations

**DOI:** 10.1155/2017/7567838

**Published:** 2017-08-07

**Authors:** Joel Caballero García, Adolfo Michel Giol Álvarez, Iosmill Morales Pérez, Nélido Gonzales Gonzales, Adolfo Hidalgo Gonzáles, Peggys Oleidis Cruz Perez

**Affiliations:** ^1^National Institute of Oncology and Radiobiology, Head and Neck Department, Havana, Cuba; ^2^Hnos Ameijeiras Hospital, Havana, Cuba

## Abstract

**Introduction:**

The paranasal sinuses mucoceles are benign expansive cystic lesions that occur rarely in the sphenoid sinus and contain mucous material enclosed by cylindrical pseudostratified epithelium.

**Objective:**

To report one case of sphenoid sinus mucocele that occurred with headache and was submitted to surgical treatment through endonasal endoscopy approach.

**Case Report:**

59-year-old male patient with history of increasing frontoorbital, bilateral, fluctuating headache and exophthalmos. There was no other associated clinical abnormality. Computed Tomography (CT) and Magnetic Resonance Image (MRI) scans confirmed an expansive mass of sphenoid sinus, suggesting mucocele. The patient was submitted to endonasal endoscopic surgery with posterior ethmoidotomy, large sphenoidotomy, and marsupialization of the lesion.

**Conclusion:**

Mucoceles of the sphenoid sinus are a very rare condition with variable clinical and radiological presentation. Surgical treatment is absolutely indicated and early treatment avoids visual damage that can be permanent. Endonasal endoscopic approach with drainage and marsupialization of sphenoid sinus, using a transnasal corridor, is a safe and effective treatment modality.

## 1. Introduction

The paranasal sinuses mucoceles are benign expansive cystic lesions that occur rarely in the sphenoid sinus and contain mucous material, enclosed by cylindrical pseudostratified epithelium [1]. The treatment is surgical and currently, endonasal endoscopic method is the modality of choice [2]. However, there are less than 200 cases reported and only few treated by endoscopic approach with different technics that include marsupialization or mucosal remotion [3]. The lack of consensus responds to their low frequency. In this article we describe the case of a patient with sphenoid sinus mucocele (SSM) and discussed the surgical technic.

## 2. Case Report

A 59-years-old male patient, with a 2-year history of increasing frontoorbital bilateral and fluctuating headache, is presented. There was no other associated clinical abnormality, loss of vision, or nasal obstruction. On admission, there was no sign of meningeal irritation. Clinical exam showed bilateral exophthalmos. Ophthalmological review and fundoscopy were normal. Eyes movements were normal and painless, and neurological examination was negative. He has no history of local radiation or surgery. Rhinoscopy was negative.

**Figure 1:**
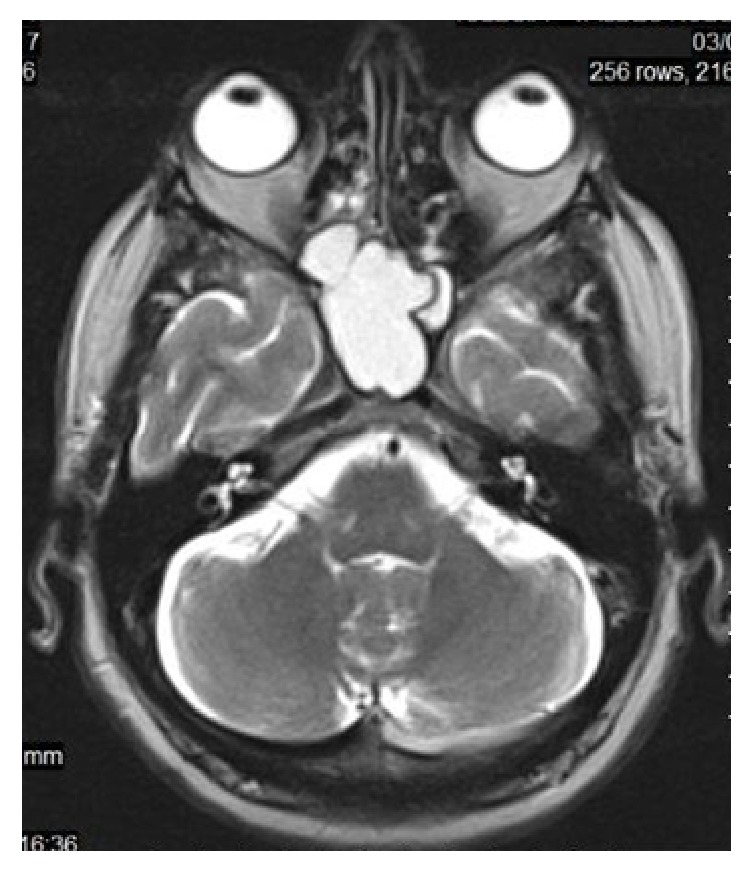
The lesion appearing hyperintense on T2-weighted RMI.

**Figure 2:**
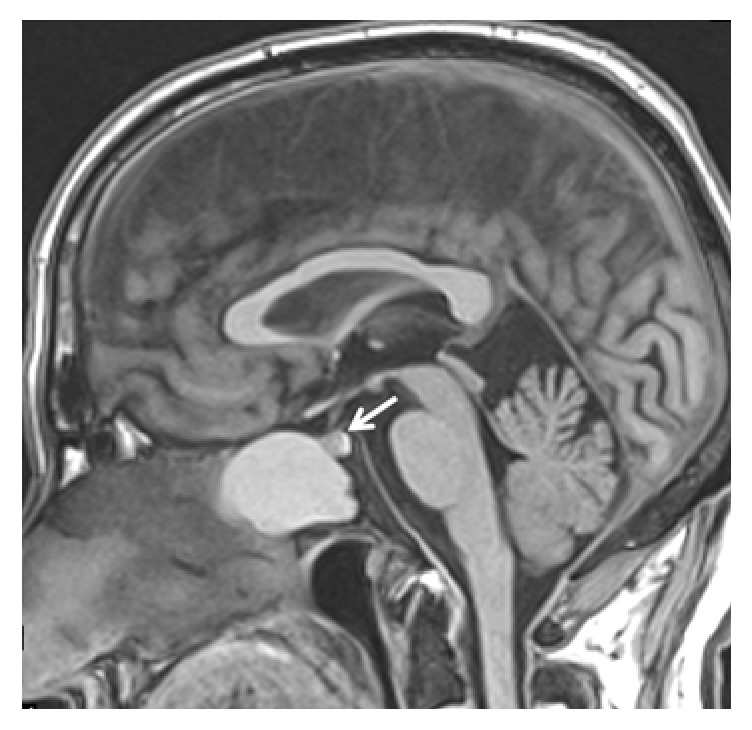
T1-weighted RMI in sagittal cut showing expansive cystic mass occupying the sphenoid sinus and posterior ethmoid. Observe the integrity of pituitary gland (white arrow).

Initially, head and paranasal sinuses CT scan was performed and revealed a large, isodense, well-defined, expansive space-occupying lesion in a midline location, centered in the body of the sphenoid bone and extended to posterior ethmoid cells. The lesion was causing marked thinning of the sphenoid sinus bony wall with bony erosion areas. There were no calcifications. MRI shows a hyperintense lesion on T1 and a hyperintense lesion on T2 weighted images. There was no suppressed fat on STIR sequence (Figure 1). In sagittal view the pituitary gland was intact and there was no sellar invasion, excluding pituitary adenoma with sphenoidal sinus extension (Figure 2).

**Figure 3:**
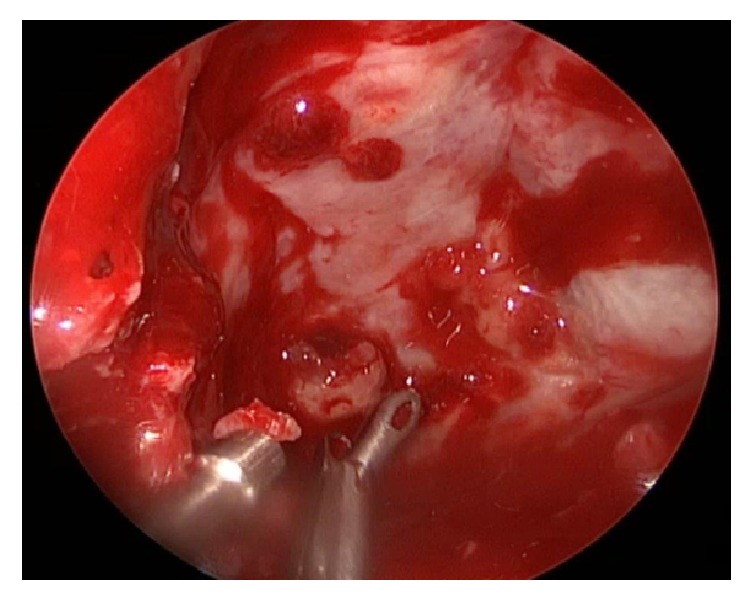
Removing of a solid lesion occupying the clival recess.

**Figure 4:**
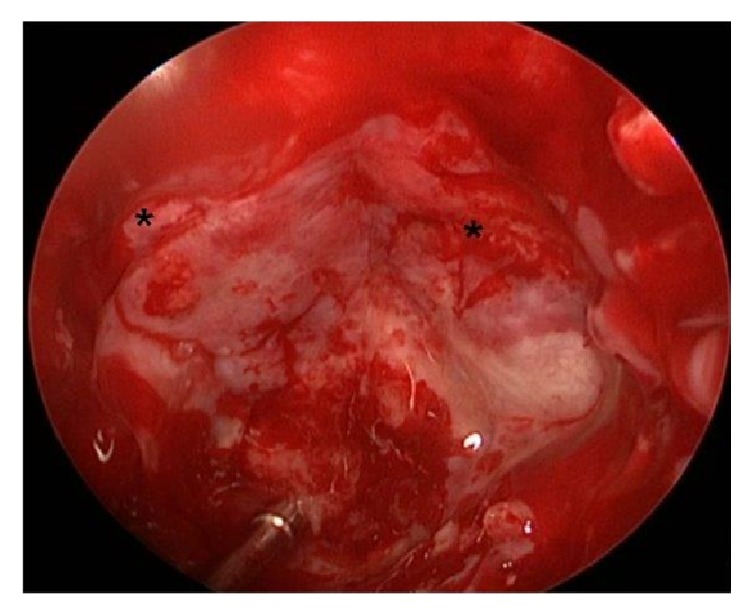
Sphenoidal sinus opened and demucosalized. Suction cannula is in clival recess. Black asterisks show lateral opticocarotid recess between carotid artery (downward) and optic nerve (upward). Observe the short intercarotid distance and the absence of prominence of the sellar floor.

The patient was submitted to endonasal endoscopic approach with large sphenoidotomy, marsupialization, and bilateral posterior ethmoidectomy. During nasal step right inferior and middle turbinates were lateralized and bilateral superior turbinates were removed. The anterior wall of sphenoid sinus was protruded forward between both middle turbinate and nasal septum. In order to make a large sphenoidotomy the posterior septum was removed. During this maneuver draining was carried out and the mucocele was empty. The sphenoidotomy showed a little solid mass, occupying clival recess that was removed. It was very thick and adherent to the walls of sphenoidal mucosa, covering an osseous erosion area, especially in opticocarotid recess region (Figure 3). Sphenoidal sinus mucosa was completely removed. Blood loss was 150 mL and surgical time was 110 minutes. The preoperative RMI shows a large intercarotid space; however, intraoperative distance between both carotid arteries was short (Figure 4).

**Figure 5:**
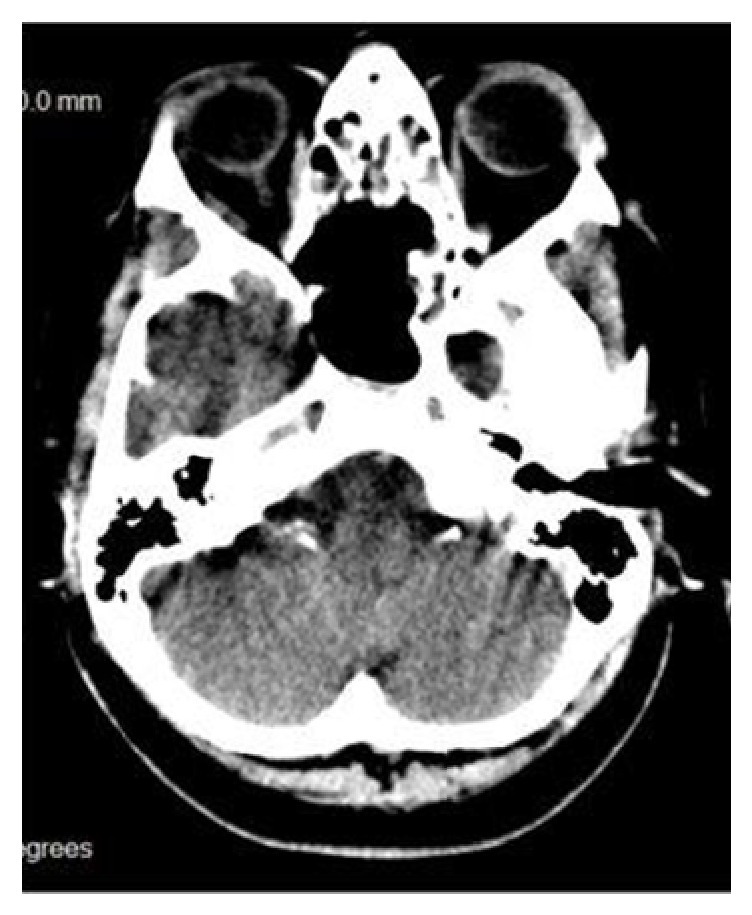
Postoperative CT scan showing the sphenoidotomy, the posterior ethmoidectomy, and the absence of lesion.

The patient was treated with ceftriaxone although culture was negative. There were no surgical or postoperative complications. The hospital stay was 3 days. Nasal irrigation with isotonic saline solution every 4 hours was indicated in the first 3 months. He underwent successful* revision *surgeries monthly during the first 6 months before surgery without nasal synechiae or other complications. He presented a complete improvement of the algic picture and postoperative CT scans showed an air-filled cavity replacing the lesion in the sphenoid body with no residual soft-tissue lesion (Figure 5). Histopathological analysis of the solid specimen confirms presence of inflammatory tissue and calcification.. He underwent 1-year postoperative follow-up.

## 3. Discussion

Mucoceles of the sphenoid sinus are a very rare condition and represent only 1% of all paranasal sinus mucoceles [1]. Almost 200 cases of sphenoidal and or intrasellar mucoceles have been reported since the first identification by Rouge in 1872 and the description by Berg in 1889 [4]. However, it represents 15 to 29 percent of all cases of isolated sphenoid disease [3].

Development mechanism of spontaneous mucocele is not clear. There are different theories: obstruction of the sinus, cystic development of embryonic epithelial residues, cystic dilatation of the glandular structures, and even an atypical form of craniopharyngioma [5, 6]. Obstruction can be due to congenital abnormalities, allergy, infection, trauma, neoplasm, radiation, or surgical intervention [1, 7].

They can be presented in an age range of 8 to 83 years (48% were 30 to 60 years old) and there is no sex prevalence. The interval between the first symptoms and diagnosis varied from 3 days to 38 years, with an average of 3.7 years [7]. They are characterized clinically by an undetermined silent initial period, followed by a period in which its expansion causes symptoms. Clinical manifestations depend on the direction of the expansion toward adjacent structures and include frontoorbital headache (87%); amaurosis (58%); oculomotor palsies (55%); nasal symptoms (38%) that include anosmia, nasal obstruction, hypoacusis, and nasal discharge [8]; endocrine disorders (3%); and panhypopituitarism (0.8%) [2]. Symptoms are often nonspecific, resulting in diagnostic delay. Fortunately the patient was presenting only headache and he received treatment before other symptoms occurred, especially visual loss. Some authors report irreversible visual loss due to sphenoidal mucocele after surgical treatment [9, 10].

Mucoceles may have variable densities on CT (most of them being hypodense) and variable signal intensities on MRI, according to their water and protein content, density, and possible infection (most of them being isointense on T1 and hyperintense on T2 weight images) and do not show contrast enhancement except some cases with thick encapsulation [1, 10]. The differentiation from simple fluid retention that is found more often than mucocele relies on the expanding character of the mucocele [11].

Differential diagnoses include necrotic primary adenoma with significant intrasellar extension, craniopharyngioma, chordoma, plasmacytoma, osteoma, osteoblastoma, basal cell and squamous cell carcinoma, rhinolitis, polyps, and fibrous dysplasia [5]. The RMI is essential for differential diagnoses.

The contents of mucoceles are usually sterile, although there may be infected, named mucopyoceles. In those cases, the most frequently isolated germs are Gram-positive organisms (*Staphylococcus aureus*,* Staphylococcus epidermidis*, and streptococci). In such cases, antibacterial therapy should be based on the organisms most likely to be encountered. In case of sterile contents high spectrum of antimicrobial drugs is recommended [7].

Surgical treatment is absolutely indicated in mucoceles [11]. The purpose of surgical treatment is to evacuate the cystic lesion, relieve the symptoms, and prevent recurrence. Currently, the endonasal endoscopic approach constitutes the modality of choice [12]. However, there are different approaches and philosophies. Some authors suggest sphenoidal ostium enlarge [1]; however, we consider that this maneuver can increase the risk of recurrence. Intranasal marsupialization of mucocele was reported as early as 1921 by Horwath [7] and recently for other authors [12, 13]. There are different ways to access the sphenoidal ostium: during a transnasal, transeptal, and transethmoidal approach. Some authors suggest transeptal approach for sphenoidal mucoceles [3]. The authors of this article consider that those approaches have some disadvantages: limited exposition that can make a complete sphenoidotomy (marsupialization) difficult and can make a posterior ethmoidectomy (in cases of ethmoidal extension like this patient) difficult. On the contrary, endonasal endoscopic approach using a transnasal route offers a wide surgical corridor and it allows a four hands two surgeons' technique, so the sphenoid sinus marsupialization, that attempts to totally exteriorize the sphenoid sinus cavity with sufficient removal of the anterior and inferior wall of the sphenoid sinus, can be do it easier. On the other hand, mucoceles develop progressively and result in resorption and even erosion of the bony walls of the sinus that can make the removal of the mucosa difficult, even with risk of vascular accident due rupture of intracavernous artery or optic nerve injury, especially during one surgeon' technique. That why we favored the 2 surgeons 4 hands' technique. Also other authors consider it the therapy of choice [7, 14]. There are few similar cases and they are less operated by means of minimally invasive surgery.

Moryiama et al. [15] did not report recurrence during follow-up periods of 10 years in any of their 47 patients treated by marsupialization. Other authors have similar results [16, 17]. However, because the mucocele can develop 15–25 years after the initial surgery, a long-term postoperative follow-up is mandatory [15].

Sphenoidal mucoceles unusually contain calcifications within their wall [18]. In this case we observe a lesion occupying clival recess and histopathology confirms the calcification. In the context of a concomitant lesion it is important to study it due to the fact that some tumor and tumor-like conditions, such as carcinoma, fibrous dysplasia, osteoma, and ossifying fibroma, might be associated [1].

## 4. Conclusion

Mucoceles of the sphenoid sinus are very rare condition with variable clinical and radiological presentation. Surgical treatment is absolutely indicated and early treatment avoids visual damage that can be permanent. Endonasal endoscopic approach with drainage and marsupialization of sphenoid sinus, using a transnasal corridor, is a safe and effective treatment modality.
